# Diagnosis of colour vision deficits using eye movements

**DOI:** 10.1038/s41598-022-11152-5

**Published:** 2022-05-11

**Authors:** Aryaman Taore, Gabriel Lobo, Philip R. Turnbull, Steven C. Dakin

**Affiliations:** 1 School of Optometry and Vision Science, The University of Auckland, Auckland, New Zealand; 2 New Zealand National Eye Centre, The University of Auckland, Auckland, New Zealand; 3 UCL Institute of Ophthalmology, University College London, London, UK

**Keywords:** Vision disorders, Colour vision

## Abstract

We set out to develop a simple objective test of functional colour vision based on eye movements made in response to moving patterns. We exploit the finding that while the motion of a colour-defined stimulus can be cancelled by adding a low-contrast luminance-defined stimulus moving in the opposite direction, the “equivalent luminance contrast” required for such cancellation is reduced when colour vision is abnormal. We used a consumer-grade infrared eye-tracker to measure eye movements made in response to coloured patterns drifting at different speeds. An automated analysis of these movements estimated individuals’ red-green equiluminant point and their equivalent luminance contrast. We tested 34 participants: 23 colour vision normal controls, 9 deuteranomalous and 2 protanomalous individuals. We obtained reliable estimates of strength of directed eye movements (i.e. combined optokinetic and voluntary tracking) for stimuli moving at 16 deg/s and could use these data to classify participants’ colour vision status with a sensitivity rate of 90.9% and a specificity rate of 91.3%. We conclude that an objective test of functional colour vision combining a motion-nulling technique with an automated analysis of eye movements can diagnose and assess the severity of protanopia and deuteranopia. The test places minimal demands on patients (who simply view a series of moving patterns for less than 90 s), requires modest operator expertise, and can be run on affordable hardware.

## Introduction

Colour vision is based on the combined responses of three classes of photoreceptor (a.k.a cones) found in the retina^1^. For individuals with typical colour vision (CVn), pigments found in the cones make each cone type sensitive to either long, medium or short-wavelengths of light. Around 4% of people—8% of males and 0.5% of females—suffer from a congenital colour vision deficiency (CVd)^2^ which impacts on their ability to perform everyday tasks and can even influence career choice^3^. CVd is commonly characterized by increased overlap in the spectral sensitivity profile of the three cone types as a result of changes in the sequence of amino acids making up the pigments^1^. For example, the two most common forms of CVd, protanomaly (red colour-blind) and deuteranomaly (green colour-blind), are associated with similarities in the spectral sensitivity of the long- and medium-wavelength sensitive photoreceptors, respectively. This in turn leads to poorer discrimination by patients making colour-judgements along the red-green chromaticity axis. Atypical photo-pigmentation typically arise from mutations of the genes encoding the cone pigments^4^. In CVd, the genes encoding red and green cone pigments are (typically) subject to unequal recombination, as a result of (a) physical proximity and (b) DNA sequence similarity (~ 96%)^5^. This unequal recombination leads to deletion of genes or to the generation of genes that encode pigments sensitive to wavelengths intermediate to the usual peak sensitivity of red and green cones.

Because everyday activities like driving rely on accurate colour vision, colour vision testing is a core activity in optometry. However, when it comes to red-green colour-vision deficits, the standard clinical evaluation (Ishihara plates) cannot accurately grade the severity or type of defect^6^. The gold-standard test for diagnosing and quantifying the severity of a CVd uses an anomaloscope^7^, but such testing is not routine in most clinics as it is time-consuming, requires high levels of operator expertise, and involves patients making a protracted series of subjective judgements of colour appearance. Such considerations limit the use of this test with children^8^. Further, detailed diagnostics are considered less useful in the absence of any *treatment* for CVd^9^ although this may not always be the case given recent developments in retinal gene therapy^10–12^.

In short, the limitations of current colour vision testing highlight the need for a simple-to-use, rapid and objective measure of functional colour vision that could be used with a broad range of patients. Here we describe our effort to produce such a test: an automated implementation of a motion-nulling test first described by Cavanagh and Anstis^13^ that exploits a link between subjective colour impression and eye movements made in response to motion.

**Figure 1:**
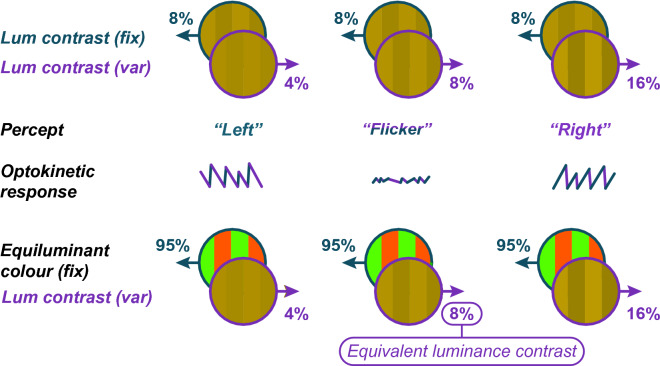
(Top row) Two superimposed gratings moving in opposite directions, with the contrast of one grating fixed (here at 8%) and the other varying. (middle rows) The observer's optokinetic response to the summed gratings, generally follows the direction of the grating that *appears* higher-contrast. When the contrasts are *perceptually matched* the observer perceives only flicker, and the motion of the fixed grating is "nulled". (bottom row) A relatively low contrast (here 8%) luminance-defined grating can null the motion of a high-contrast equi-bright colour grating.

Although it has been proposed that our sense of motion is driven exclusively by luminance-sensitive mechanisms^14^ we do experience a weak sense of motion of equiluminant moving stimuli defined only by spatial modulation of chromatic information^15–17^. The contribution that colour makes to our sense of motion in more realistic stimuli (i.e. that are defined by *both* colour and luminance*)* has been explored extensively using motion nulling^13^ (top row, Fig. 1). These paradigms use superimposed patterns moving in opposite directions. If the contrast of one component is fixed and the contrast of the other is varied, then the observers’ percept will generally follow the direction of the higher-contrast stimulus. Perceived direction can then be estimated by either (a) having observers make a two-alternative-forced-choice (2AFC) classification of perceived direction as either “left” or “right”, or (b) by analysing involuntary tracking eye movements (*optokinetic response*). When components are contrast-matched then the stimulus appears to flicker rather than drift (a) causing observers to guess its direction and (b) eliciting no coherent eye movement response. At this point the fixed-contrast motion is said to have been nulled^18^.

While Levinson and Sekuler’s original paradigm used only luminance-defined stimuli, nulling occurs between stimuli defined along completely different dimensions when *perceived strength of movement is matched.* Of note, Cavanagh et al.^13^ showed that the minimum contrast of a luminance-defined grating required to null the motion of a colour-defined grating (bottom row, Fig. 1) is a simple way of quantifying the contribution of colour to motion. This contribution—termed *equivalent luminance contrast* (*C*_Eq_)—is around 8% luminance contrast (bottom row, Fig. 1) in participants with typical colour vision (CVn), but can be much lower in patients with red or green colour vision deficits^13^ (hereafter referred to as protanopes or deuteranopes respectively). This finding is consistent with work using other methods to estimate the contribution of colour to motion e.g. by quantifying the change in motion aftereffect that arises when chromatic modulation is added to a luminance-defined adapting display^19,20^.

Determining *C*_Eq_ is possible only when the two colour components of the colour stimulus appear to have equal brightness (or are *equi-bright*). For CVn and Cvd-deuteranopic observers, the red/green components of such a stimulus will be close to physically equiluminant, but this is not the case for protanopes. To determine when red and green appear equi-bright one can again use a nulling paradigm where, now, red and green superimposed gratings move in opposite directions. In this case protanopes need a high ratio of red to green for motion to be nulled.^21^ The results of these two nulling procedures—the red/green equi-brightness level and *C*_Eq—_were used by Cavanagh et al.^13^ to determine colour vision status. Cavanagh et al.^13^ later co-varied both the luminance of a nulling grating and the R/G balance of the coloured grating to simultaneously determine *C*_Eq_ and the red-green equi-brightness point in one run. This is the approach we adopt here.

Although the Cavanagh et al.^13^ study relied on observers’ subjective report of direction to estimate nulling-contrast, the same group^21^ has also measured eye movements (optokinetic nystagmus; OKN) in response to coloured bi-directional stimuli, and used OKN to drive a nulling paradigm. OKN is a class of involuntary eye movement made in response to large-field visual motion, consisting of a sequence of periods of slow-tracking eye movements in the direction of the stimulus interspersed with rapid corrective saccades in the opposite direction. Such eye movements are mediated by partially shared brainstem and spinal pathways^22^ and serve to minimise retinal slip^23^. Plotting the horizontal position of the eye (*y*-axis) against time (*x*-axis) leads to a characteristic “saw-tooth” pattern, the slope of which varies with direction of the slow-tracking movement (middle of Fig. 1). Critically, the direction of the tracking-phase of the participants’ OKN response closely matches their subjective report, a finding that has been confirmed in both human and non-human primates^24–26^. Because the strength of OKN is generally determined by the visibility of moving stimuli, and visibility can be manipulated along a variety of visual dimensions, OKN has proven to be a flexible technique for assessing visual function such as acuity^27^ contrast sensitivity^28^, visual field loss^29^ and refractive error^30^. Compared to perceptual report, OKN provides a more objective measure, requiring minimal compliance (typically only passive viewing of a series of movies).

Other studies have used eye movements to measure chromatic sensitivity in humans^31,32^ and in non-foveate vertebrates^33^. Results indicate that purely chromatic stimuli elicit OKN^34,35^ provided the grating is of a sufficiently high contrast to support discrimination of its direction; the contrast required is considerably higher than for a luminance stimulus^36,37^. In the wider OKN literature, instructing participants to “attempt to fixate” (*stare-OKN*) generally results in low-amplitude and high-frequency nystagmus in comparison to the behaviour observed when participants are instructed to “follow the stimulus” (*look-OKN*) which leads to low-frequency and large-amplitude nystagmus^38,39^. The use of look-OKN paradigms which encourage active tracking of the stimulus leads to estimates of higher chromatic sensitivity^40^. For example Crognale and Schor^41^ recorded voluntary pursuit and involuntary stare-OKN eye movements made by observers in response to drifting equiluminant stimuli. They note irregular stare-OKN responses to purely chromatic stimuli, compared to a reliable look-OKN responses to the same stimuli.

Other non-visual factors can influence optokinetic response. The magnitude of OKN elicited can vary between participants and even across sessions for the same participant^42^. Age also has an impact on OKN, with studies showing a decrease in OKN gain of 6 to 18% (relative to a baseline of around 80%) in individuals over 50^43,44^. In addition, fatigue can reduce saccadic velocity in the OKN response, whereas administration of a stimulant such as caffeine can increase OKN gain^45^. Finally, increased attention leads to higher gain and frequency of OKN ^43,46,47^. In short, while within-subject changes of OKN response can provide an objective proxy of their perception, such between-subject and non-visual factors make it challenging to use the optokinetic response to group individuals who share a similar perceptual experience (e.g. as a result of a CVd).

With these considerations in mind, and seeking to improve the reliability of the oculomotor response across participants, we encouraged our participants to follow the stimulus using a “look-OKN” paradigm. As a result, both optokinetic reflex and active tracking of the stimulus (a.k.a. smooth pursuit) contribute to the response we measured. Following earlier work^31^ we refer to this collective pattern of eye movement response as directed eye movements (DEM). Our study builds upon Cavanagh et al.^13^ previous work, by developing an objective test that uses DEM (instead of subjective responses) to accurately measure the type and severity of colour vison deficiency. We use an infrared eye tracker to measure eye movements in response to bi-directional coloured stimuli, with automated analysis of those eye movement data to objectively quantifying strength of DEM. Plotting DEM-gain as a function of red-green luminance-balance allows us to make an objective estimate of both equivalent RG-brightness (red-green luminance-balance) and equivalent luminance contrast *C*_Eq_. We use these pairs of measures to train a classifier to distinguish CV-status and compare results to a gold-standard clinical colour vision assessment.

## Methods

We ran three colour vision tests on observers with and without congenital CVd. The first was the gold standard Neitz Anomaloscope. The second was the clinical standard 14-plate Ishihara. The third was our updated Cavanagh/Anstis test that estimated both red-green equiluminant point and equivalent luminance contrast estimated from participants’ directed eye movements to motion-nulling stimuli.

### Participants

We recruited 34 participants (17 females, 17 male, 17–65 years), of which 23 were normal trichromat controls, 9 were deuteranopes and 2 were protanopes (see Appendix A for patient details). We targeted recruitment of participants with red-green colour vision deficiencies or normal trichromatic colour vision. Due to the strobing appearance of some stimuli, participants with epilepsy were excluded. The experimental protocols and procedure were approved by the University of Auckland Human Research Ethics Committee. The protocols and procedure complied with the Declaration of Helsinki, and informed consent was obtained from all participants prior to the experiment.

### Apparatus

**Figure 2:**
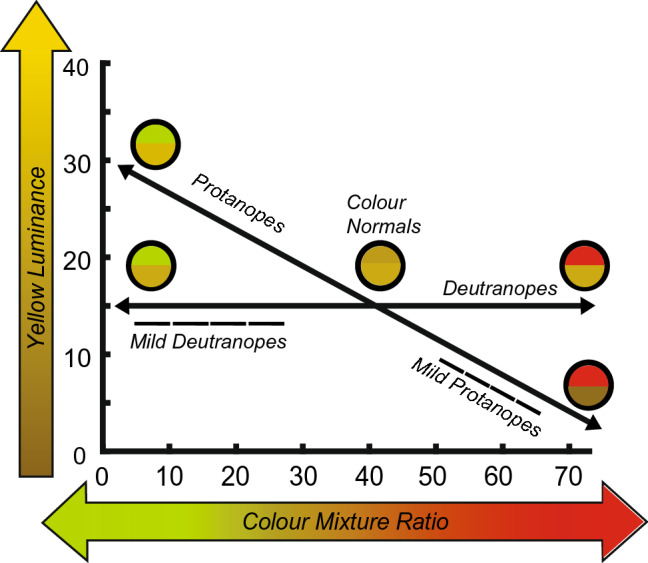
Expected results from the anomaloscope for different CVd groups. The x-axis shows the ratio of red-green in the reference ranging from 0 (pure green) to 73 (pure red). The y-axis indicates the luminance of the yellow test scaled from 0 (black) to 40 (maximum brightness)—that matched the corresponding red-green test. Lines are labelled with the corresponding CV category. Because the red and green primaries of the anomaloscope lie along the red-green axis, the test captures the colours readily confused by dichromats. As a result, extreme dichromats can match the yellow test to red-green references spread across the entire colour mixture range—solid arrows. CVn observers on the other hand see contrast between colours and will match only at near-equal ratios of red and green (40–50). Mild dichromats—dashed lines being less sensitive to either red or green, require a greater amount of that colour to make a luminance match, thus matching values on either side of the CVn range.

A Type-I Neitz anomaloscope (Model OT-II) was used to perform a standard diagnosis of red-green CVd by having the participants adjust the luminance of a yellow test to match the appearance of the range of red-green mixed reference colours^7^. Plotting the results as in Fig. 2, the position and slope of matched values indicates the type of CVd, whereas the length of the matching range quantifies the severity of CVd.

For the eye tracking tasks we presented stimuli and recorded eye movements using a Windows 10 PC laptop (ROG Zephyrus M) fitted with a Tobii 4c eye tracker^48^. The laptop had a 15.6 inch (1920 × 1080 pixels), LCD IPS display operating with a 120 Hz refresh rate. The screen was viewed under standard room lighting (Illuminant D65) and at approximately 40 cm without head/chin support. The luminance of RGB components of the screen was linearised in software using measurements made with a Konica Minolta LS-110 photometer. Stimuli were created in MATLAB (MathWorks, NA) using elements of the PsychToolbox^49^.

A Tobii 4c eye tracker recorded a series of infrared images of the user at 90 Hz to (a) localize the face and (b) estimate monocular and binocular gaze point on-screen (*Active Display Gaze Point* in the Tobii Pro software framework). Despite being a consumer grade eye tracker, the quality of eye tracking is sufficient to capture OKN, as demonstrated in related research^50^. The experiment was performed without any chin or headrest, although participants were instructed to attempt to maintain a constant head position. Note that in our pilot testing, we measured almost identical responses regardless of whether the head was fixed or not.

### Stimuli

**Figure 3:**
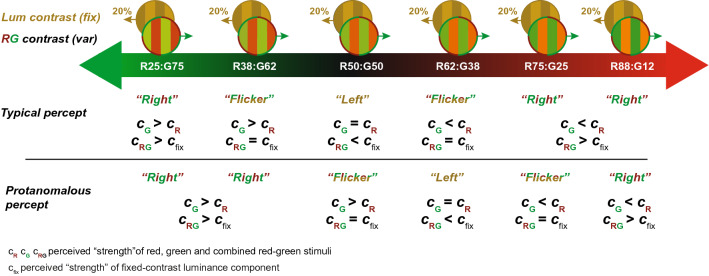
Perception of direction in the eye-tracking test. (Top row) Stimuli are comprised of a fixed-contrast (20%) yellow luminance grating added to a R-G coloured grating. (Middle row) When a yellow grating is superimposed on an equiluminant RG grating (R50:G50), a CVn observer will report the direction of the yellow component, because its contrast exceeds *C*_Eq._ Increasing the luminance of either the red or green component first nulls the fixed-contrast component (leading to flicker) and then exceeds it (at which point observers report direction of the R-G grating). (Bottom row) By comparison, an observer with protanomalous colour vision who perceives red as weaker than green—produces responses shifted right along the stimulus axis. Note how the observers require more red (here R62:G38) to achieve equi-brightness.

Stimuli were composed of superimposed pairs of vertical sine-wave gratings moving in opposite horizontal directions (Fig. 3 top row, and Video 1). Gratings were defined either by modulations of luminance (generated by in-phase spatial modulation of red and green channels) or of chromaticity (generated by anti-phase spatial modulation of red, and green channels). All gratings had a peak spatial frequency of 0.5 cycles per degrees (i.e. 1 cycle of a sinewave grating for every two degrees of visual angle) and subtended 46.54° by 27.26° presented fullscreen on the laptop monitor. These parameters were selected based on previous studies showing that such stimuli induce robust OKN^28,51^. In pilot testing we noted that the stimulus factor predominantly responsible for driving variability in DEM response was grating speed. We therefore varied this parameter experimentally between 4, 8 or 16 deg/s, to determine which speed led to optimal classification of test from controls. During tesing, the contrast of the luminance-defined grating, *C*_*Fix*_, was maintained at 10% or 20%, while the mixture of red *M*_*Red*_ and *M*_*Green*_ varied from 25 to 75%, to cover the range of red-green equibrightness-points of participants.

To generate drifting sinusoidal gratings the luminance of the red ($$L_{r } )$$ and green ($$L_{g } )$$ channels were set using:

1 $$\begin{array}{*{20}c} {L_{{{\text{R}} }} \left( {x,t} \right) = L_{{{\text{mean}} }} + L_{{{\text{range}}}} \frac{{C_{{{\text{Lum}} }} {\text{ cos}}\left( {2\pi f_{s} x + 2\pi f_{t} t} \right) + R {\text{cos}}\left( {2\pi f_{s} x - 2\pi f_{t} t} \right)}}{4}} \\ \end{array}$$

2 $$\begin{array}{*{20}c} {L_{{{\text{G}} }} \left( {x,t} \right) = L_{{{\text{mean}} }} + L_{{{\text{range}}}} \frac{{{ }C_{{{\text{Lum}} }} {\text{cos}}\left( {2\pi f_{s} x + 2\pi f_{t} t} \right) + \left( {1 - R} \right) {\text{cos}}\left( {2\pi f_{s} x - 2\pi f_{t} t - \pi } \right)}}{4}} \\ \end{array}$$

where $f_{s}$ and $f_{t}$ are the spatial and temporal frequency respectively, *L*_mean_ is the mean luminance of the display (36.0 cd/m^2^), and *L*_range_ is the luminance range (± 35.6 cd/m^2^). Perception of stimulus direction will vary as the ratio of red-to-green luminance changes. Figure 3 depicts the typical percept for a CVn (middle row) and a protanomalous trichromat (bottom row).

Participants were presented with six 75 s long movies in total, each displaying a single combination of component-grating speed and fixed luminance contrast (4 Hz + 10%, 4 Hz + 20%, 8 Hz + 10%, 8 Hz + 20%, 16 Hz + 10%, 16 Hz + 20%). Each movie comprised forty 2.5 s trials. Direction of grating (left or right) and the proportion of red (25%, 37.5%, 50%, 62.5%, 75%) were shuffled across trials, to minimise the build-up of optokinetic aftereffects. Each combination of direction and proportion of red of the coloured grating was repeated four times. Participants were instructed to follow the stimulus if it felt natural. Eye movements were scored using the procedure described in the next section below.

### Analysis: quantifying directed eye movements (DEM)

To quantify the direction and magnitude of DEMs induced by our stimuli we adapted an approach for measuring contrast sensitivity using the optokinetic response^28^. We first pre-processed eye tracking data, breaking the sequence up into fragments punctuated by blinks. Blinks were signalled when instantaneous pupil-diameter deviated from the median pupil size by more than 3 times the mean absolute deviation of the pupil-diameter. Eye position data collected during—or within 33 ms of the onset or offset of—a blink, were discarded. The remaining eye-position data were used to calculate an *eye-velocity threshold* which was used to classify instantaneous estimates of horizontal eye velocity as either saccadic or tracking movements. The threshold was set such that it would maximise the distance travelled by the eye ($$D)$$, assuming DEMs/optokinetic nystagmus in the stimulus direction ($$\theta$$). $$D_{\theta }$$ then, was the sum of all eye-movements classed as tracking ($$T_{\theta }$$) in the same direction as the stimulus, and saccades ($$S_{{\theta + {\uppi }}}$$) in the opposite direction of the stimulus motion:

3 $$\begin{array}{*{20}c} {D_{\theta } = T_{\theta } + \,S_{{\theta + {\uppi }}} } \\ \end{array}$$

$D_{\theta }$ quantifies strength of DEM in degrees and a similar calculation was performed for the opposite direction to give $D_{{\theta + {\uppi }}}$. The calculated velocity threshold aimed to maximise $C_{\theta }$, the ratio between $D_{\theta }$ (consistent with DEM in the direction of the coloured grating) and $D_{{\theta + {\uppi }}}$.

4 $$\begin{array}{*{20}c} {C_{\theta } = \frac{{ D_{\theta } }}{{D_{\theta } + \,D_{\theta + \pi } }}} \\ \end{array}$$

The measure we use to characterise DEM strength—*DEM-gain*—is like $D_{\theta }$ except it is calculated only using tracking velocity. It is the ratio of mean tracking velocity ($T_{\theta }$) to mean stimulus-velocity.

### Analysis: estimating equibrightness and equivalent luminance contrast

**Figure 4:**
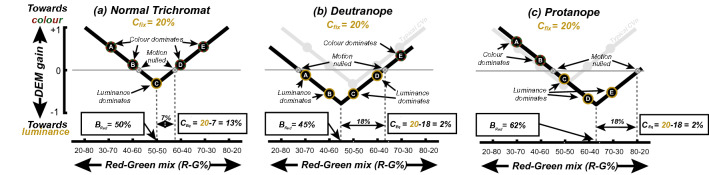
Schematic DEM gain response for (**a**) CVn, (**b**) CVd (deut) and (**c**) CVd (prot) participants viewing variable-contrast colour gratings in the presence of a fixed-contrast (20%) luminance grating. (**a**) Expected DEM-gain plot against red/green colour mix of the coloured grating. Points A and E show a strong positive response (in the direction of the coloured grating) when the colour mix is dominated by either red or green. When colour-mix leads to equi-brightness (point C; quantified as *B*_Red_), DEM is poorly driven by the coloured grating and switches to the direction of the luminance-defined grating. At points marked $\otimes$ colour and luminance gratings elicit similar DEM in opposite directions, leading to motion nulling. (**b**) and (**c**) With a CVd (deut) and CVd (prot) observer the tip of the “V-function” is shifted horizontally relative to equiluminance (50–50); e.g. (**b**) leftwards for a deuteranope and (**c**) rightwards for a protanope. Boxed equations in (**b**) and (**c**) explain how equivalent luminance contrast (*C*_Eq_) is estimated from shifting of motion null-points caused (here) by the V-function shifting downwards (Video 2).

Plotting DEM-gain against the red-green luminance-balance (Fig. 4a) yields a V-shaped function which has a minimum at the equi-brightness point and (normally) crosses the zero-gain line at two points (the two red-green mixtures leading to motion nulling, as discussed in Fig. 3). We fit these data using a standard V-function with three free parameters:

5 $$\begin{array}{*{20}c} {R = S\left| {M_{{{\text{red}}}} - B_{{{\text{red}}}} } \right| + A} \\ \end{array}$$

where *R* is the predicted DEM-response, and *M*_red_ is the red component of the red-green colour mixture of the coloured grating. The three fit parameters are: *B*_Red_ (the red-green mix that minimizes *R,* i.e. the equi-brightness point), and *A* and *S* are offset and scaling parameters respectively. From V-functions fit to each participant’s six data-sets (2 fixed luminance contrasts × 3 stimulus speeds) we record the equi-brightness point *B*_Red_ and calculate *C*_Eq_, the equivalent luminance contrast. *C*_Eq_ is estimated based on the zero-crossings of the V function (inferred *M*_red_ levels that would lead to motion nulls). By setting *R* = 0 in Eq. 5, and rearranging we see that the zero crossings arise at:

6 $$\begin{array}{*{20}c} {B_{{{\text{red}}}} \pm \frac{A}{S} } \\ \end{array}$$

*C*_Eq_, then is defined as difference between the fixed luminance contrast *C*_fix_ and the distance of the nulls from *B*_Red_ i.e.

7 $$\begin{array}{*{20}c} {C_{{{\text{Eq}}}} = C_{{{\text{Fix}}}} - \frac{A}{S} } \\ \end{array}$$

Figure 4b,c illustrate how the V-shaped function is shifted for a deuteranope and protanope observer. These observers have both atypical points of equi-brightness and experience weak motion in equi-bright stimuli, resulting in lower *B*_Red_ and *C*_Eq_ values respectively.

## Results

**Figure 5:**
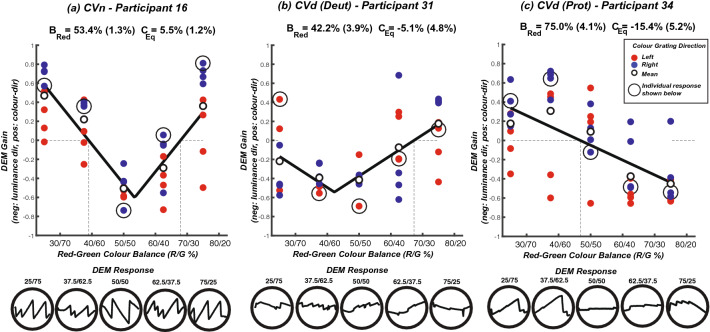
Representative ‘V-functions’ for a 20% fixed luminance-contrast component drifting at 16 deg/s. Plots show data from (**a**) a participant with normal colour vision, (**b**) a deuteranope and (**c**) a protanope. In total 8 trials for each red-green condition were carried out: in 4 trials the coloured grating moved left (red symbols), and in 4 it moved right (blue symbols). The V function was fit to the average DEM-gain across trials (small open symbol). Note the left/right shifting of the V functions in (**b**) and (**c**), indicating a shift in the red-green equiluminant point. Note also that in (**b**, **c**) the end-points of the V-functions are shifted downwards indicating a weak contribution of colour to these participants’ perception of motion^13^. The raw eye-position plots at the bottom of the figure are for conditions corresponding to the circled datapoints in the graphs.

Representative plots of DEM-strength versus red-green colour balance are shown for normal and colour deficient observers in Fig. 5. DEM-strength is signed positive or negative for whether the tracking-phase of DEM was consistent with the direction of the colour or luminance component, respectively. In Fig. 5a the participant with normal colour-vision exhibits a robust DEM response in the direction of the coloured grating at both extreme red/green luminance-balances. The luminance contrast leading to nulling of colour-motion (*C*_Eq_ = 14.5%) is lower than the fixed luminance (20%), to yield an equivalent luminance contrast of 20.0–14.5 = 5.5%. As we approach the equi-brightness point (53.4%), note the switch to a strong negative DEM response in the direction of the luminance-defined grating.

For CVn observers the V-function is relatively symmetrical around the physical equiluminance point (50–50 on the x-axes). For CVd observers, equi-brightness and the “V” function is shifted towards the defective colour. A more severe CVd leads to responses being dominated by the luminance-defined grating, shifting the “V” downwards and reducing *C*_Eq_ accordingly. Horizontal and vertical shifting of the V function is evident in Fig. 5b,c. The end-points of the V-function for both CVd responses are shifted towards the zero line as colour contributes less to their motion response (*C*_Eq_ = − 5.1% for the deuteranope and *C* = − 15.4% for the protanope*)* leading to a shallower V-function*.*

This shifting could lead to a negative *C*_Eq_ (as seen in Fig. 5b,c) consistent with the colour-grating driving the motion response *in the opposite direction to itself.* We consider two explanations for this finding. First it could be noise; our paradigm lacks the resolution to precisely determine *C*_Eq_ since it only presented coloured stimuli at a series of fixed possible red-green mixtures. A second suggestion is that this outcome could arise from aliasing of the stimulus in the periphery^52–54^.

**Figure 6:**
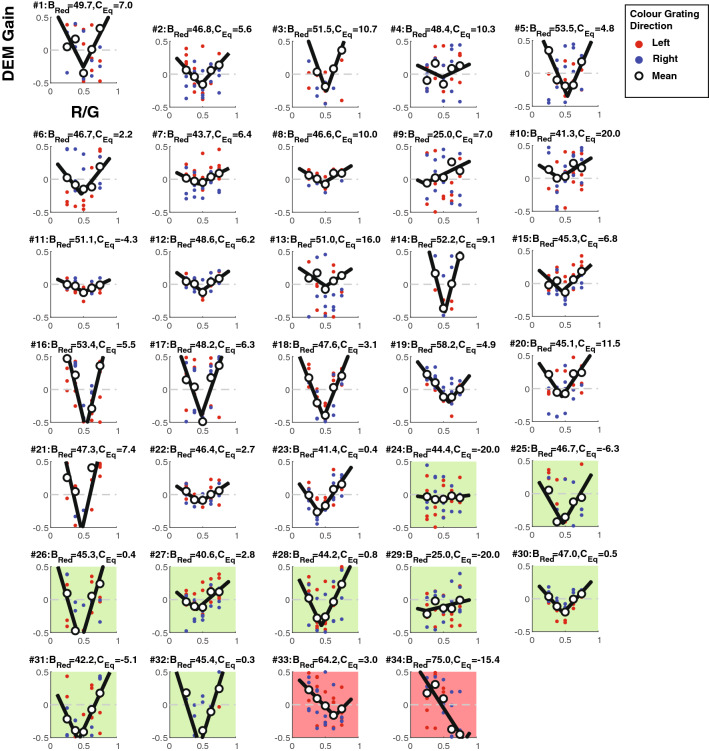
DEM gain (signed for direction of colour component as Fig. 5) measured with a 20% fixed luminance-contrast component drifting at 16 deg/s. Data have been plot on common axes to allow visual comparison of the ‘V’ fits; some data lies outside the axis range. Plots in green, red and white panels show data from deuteranopes, protanopes, and CVn observers respectively. Red and blue symbols show gain-estimates from trials when the coloured grating moved left or right respectively, with the mean-gain across trials indicated by open symbols.

Figure 6 shows the pattern of response across all participants observing stimuli moving at 16 deg/s with a fixed luminance contrast of 20%. Note the high levels of variation in DEM gain (across trials at a given stimulus level) and in overall pattern of DEM gain (although data are generally well captured by the V-fit) between participants belonging to the same colour vision group. In particular participants with the same CV status exhibit wide variation in the depths of the fit ‘V’ function. This is not unexpected given that gain in one component of their DEM-response—OKN—can vary due to a range of factors including but not limited to observer age^44^, attention^47^, fatigue^45^, and the instructions received^39^. However, because the ‘V’ fit is dependent on the *relative* DEM strength across R-G stimulus levels, absolute DEM strength across participants should not influence fits greatly. Rather *inconsistency* in DEM gains did influence the V-function fit (Fig. 6, #9, #24, #29), generally producing “shallower” fits that were typically different to the characteristic ‘V’ fits of other participants belonging to the same colour vision group.

While both protanopes (red panels) had V-functions shifted to the right, most deuteranopes (green panels) had V-functions that were symmetrical around the physical-equiluminance point, like those of observers without a CVd. As such, while *B*_Red_ best separated protanopes from CVn (red cells Table 1), *C*_Eq_ better separated deuteranopes from CVn (green cells Table 1). A summary of the group differences when compared using a Wilcoxon rank sum test is noted in Table 1. Because age too can affect the OKN response^55^**,** we also include additional comparisons between groups based on age (specifically, 50 years of age and under and over 50 years of age based on previous literature^44^) in Table 1. We report that for individuals 50 years and under and individuals over 50 years, the differences in *B*_Red_ for CVn and CVd (Prot) are significant. Whereas for *C*_Eq_, only individuals who are 50 years and under show significant difference between CVn and CVd (Deut).

**Table 1:**
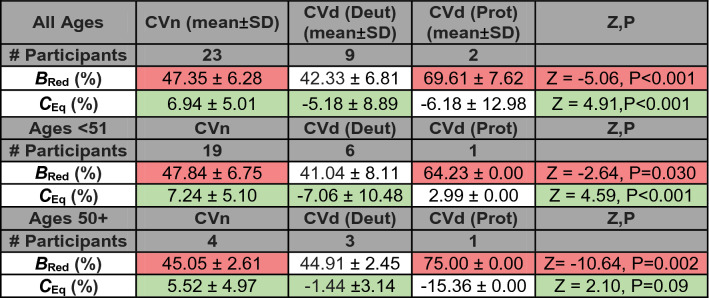
Comparison between mean *B*_Red_ and *C*_Eq_ values for colour vision groups (top 4 rows) for participants of all ages, (middle 4 rows) for participants of age 30 and under and (bottom 4 rows) for participants over 30 years of age.

Red cells highlight the comparison in *B*_Red_ between normal trichromats and protanopes. Whereas the green cells highlight the comparison in *C*_Eq_ between normal trichromats and deuteranopes. Note that for all age filters, the differences in *B*_Red_ for CVn and CVd (Prot) are significant and for all age filters (except for 50 years and over) the differences in *C*_Eq_ for CVn and CVd are significant.

As shown in Table 1, participants belonging to the same colour vision group had substantial variation in their *B*_Red_ and *C*_Eq_ values as indicated by the values’ standard deviations (SD). For CVn (of all ages), the SD of *B*_Red_ was equal to 6.28%, and for CVd (of all ages), the SD of *C*_Eq_ was equal to 8.95%. To reduce variability, we selected parameters from the “deeper” V function (quantified using the magnitude of the scaling parameter) of the two C_fix_ levels for the same stimulus speed condition. We elected to do this because visual inspection of our data suggested that some parameters from some conditions were unreliable (usually as a result of noisy DEM responses that were fit to “shallow” V functions) and by averaging them across *C*_fix_ conditions, which is what Cavanagh and Anstis did, we would likely compromise the categorisation accuracy of the system. Our process lowered the variability of measures within participants belonging to the same colour vision group, decreasing SD of *B*_Red_ for CVn (σ = 6.28% to 2.93%) and of *C*_Eq_ of CVd (σ = 8.95% to 3.54%). These better matched the low variability reported by Cavanagh and Anstis^13^ (abbreviated CA91); SD of 3.44% (for CVn for *B*_Red_) and 0.72% (for CVd for *C*_Eq_) for their most optimum test condition that best separated colour vison groups (4 deg/s).

**Figure 7:**
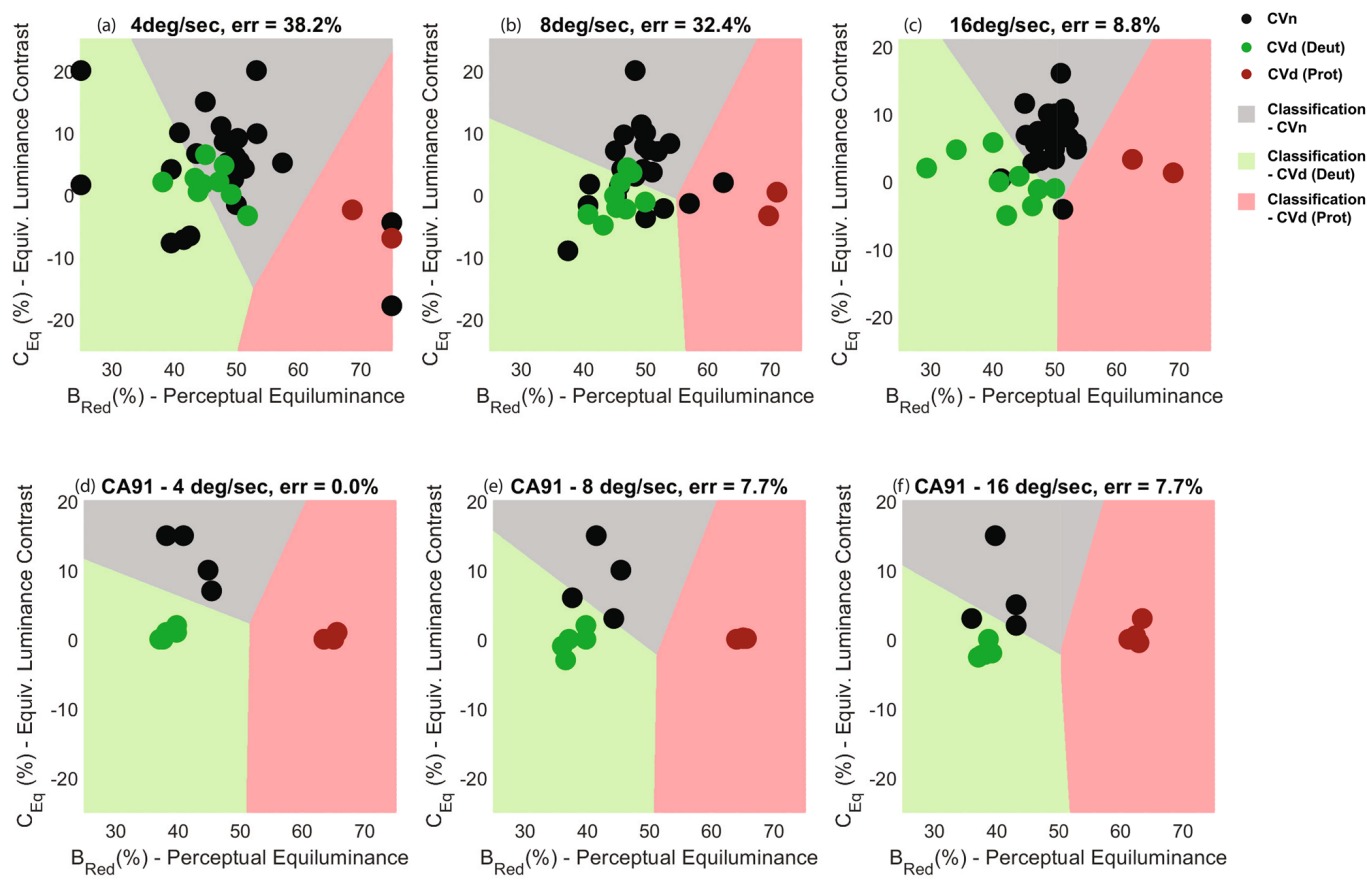
Equibrightness plot against equivalent luminance contrast for (**a**–**c**) our 30 participants and (**d**–**f**) Cavanagh and Anstis’ participants, run in the three speed conditions. Boundaries of the coloured regions were derived using a K-means algorithm that sought to best separate the three groups. Error rates indicate the percentage of mis-classified participants.

A scatterplot of individual estimates of equi-brightness against equivalent luminance, derived in this way is shown in Fig. 7.

We next considered if we could use these data to reliably determine the colour vision status of our observers. An unsupervised machine learning algorithm (K-means clustering)^56^ partitioned participants into 3 clusters in which each participant belonged to the cluster with the nearest mean (Fig. 7a–c). K-means is an iterative process that requires no labelled data. It instead initialises K centroids (where k = number of expected clusters) at distinct given locations (x,y) and moves each centroid to the average of the data-points nearest to it. This is repeated until the centroid assignment no longer changes. Based on previous findings^13^, the initial centroid positions were set to *B*_Red_ = (32.5, 50, 62.5) and *C*_Eq_ = (0, C_fix_, 0) respectively. Our results indicated that tests conducted with 16 deg/sec stimulus had the highest sensitivity (90.9%) and specificity rate (91.30%). For comparison, Fig. 7d–f plots the estimates of CA91 participants, partitioned using the same K-means algorithm.

We note the similarity of results from our best-performing test-condition (Fig. 7c) and those of CA91’s (Fig. 7d). For example, deuteranopes in both studies reached equi-brightness at somewhat more greener light (CA91: *B*_Red_ = 38.5% vs Ours: *B*_Red =_ 41.63%), whereas protanopes required a lot more red light to experience the same luminance contrast (CA91: *B*_Red_ = 64.8% vs Ours: *B*_Red =_ 65.78%). Likewise, colour deficient participants in both studies showed lower equivalent luminance contrast (CA91: *C*_Eq_ = 0.56% vs Ours: *C*_Eq_ = 0.60%) compared to CVn (CA91: *C*_Eq_ = 11.75% vs Ours: *C*_Eq_ = 6.73%).

**Figure 8:**
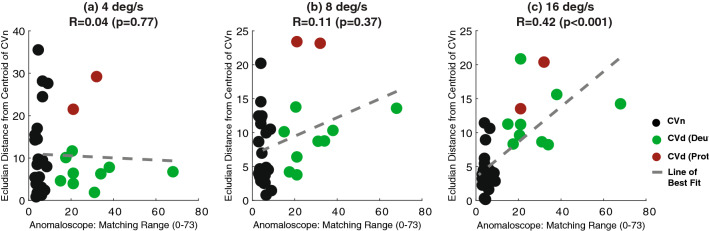
Individual estimates of matching range taken from the anomaloscope test (Appendix A), plot against individual estimates of Euclidean distance from the CVn centroid, for the three speed conditions. Larger matching ranges in the anomaloscope, and larger Euclidean distances are both indicative of more severe CVd. Line of best fit is derived using linear regression.

In addition to this, it was found that the euclidean distance between a participant’s *B*_Red_ and *C*_Eq_ and that of the centroid of the CVn cluster (found using the K-means algorithm) acted as a measure of his/her CVd severity. However, only at the highest speed condition did this measure significantly correlate (using the Kendall Rank correlation test) with the severity measure made using the anomaloscope (Fig. 8).

### Response bias

We note that CA91^13^ achieved better separation between colour vision groups when participants were presented with stimuli drifting at slower speeds (4 deg/s). They report that equivalent luminance contrast of controls was reduced as the temporal frequency was increased, leading to increased misclassification of CVn as CVd (deut), and vice versa. Despite including a small sample of only four CVn’s (of which three’s *C*_Eq_ decreased with speed) CA91’s results are consistent with previous work showing reduced sensitivity to colour at higher temporal frequencies^36,57^. In contrast we found that the equivalent luminance contrast of controls modestly increased with grating speed and the higher speed conditions produced data that *better* separated our participants into distinct colour vision groups.

**Figure 9:**
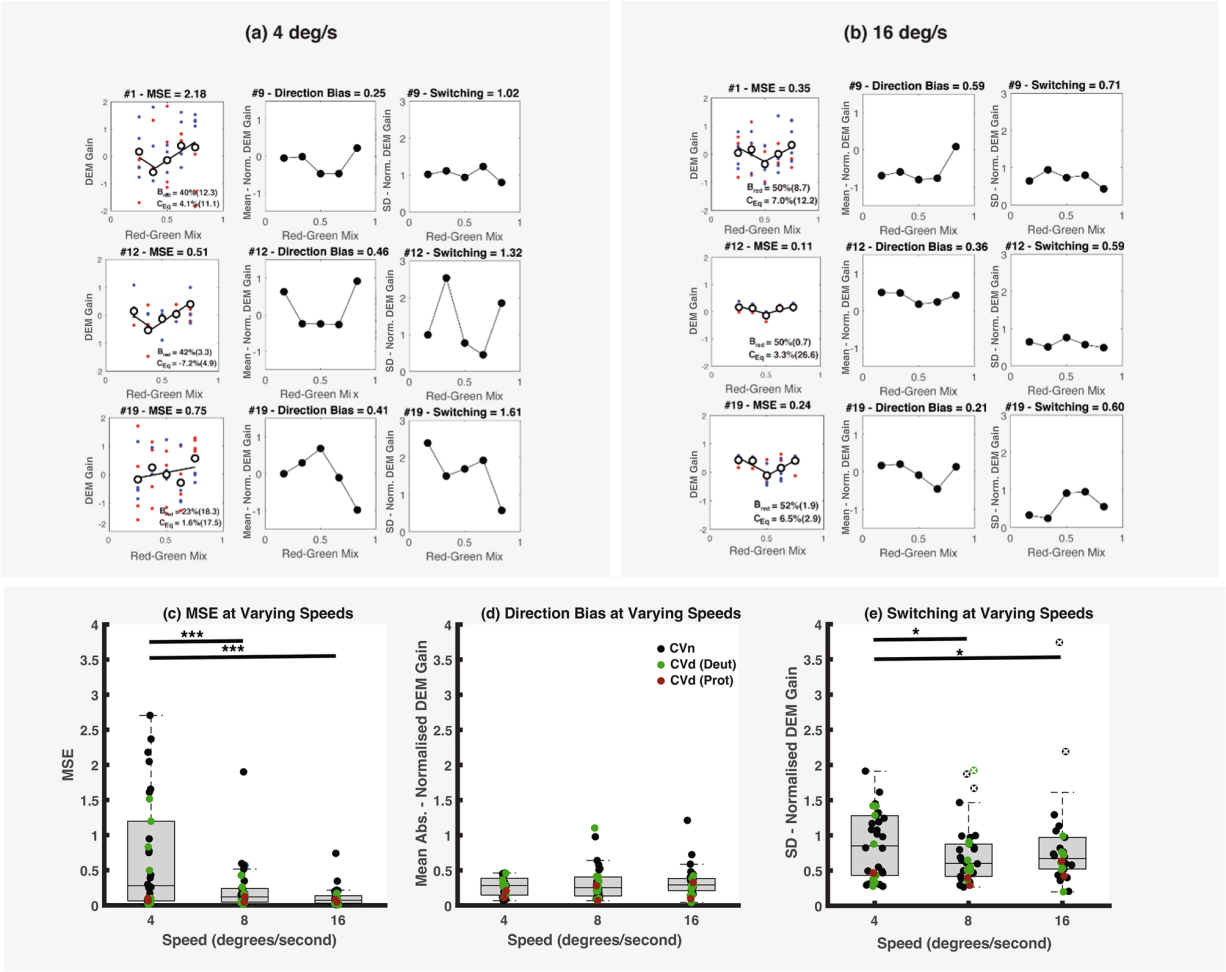
(**a**, **b**) ‘V’ functions of the 3/6 CVn participants that were misclassified (based on results from the 4 deg/s conditions) for both (**a**) 4 and (**b**) 16 deg/s conditions. Stimuli moving at 4 deg/s elicit higher but inconsistent DEM gain (as measured by the MSE). More consistent but lower DEM gains are elicited by faster stimuli which leads to more robust fits of the ‘V’ function centred around *B*_Red_ = 50%. (**c**) MSE of the V-fit across speed conditions. The mean MSE at both 8 and 16 deg/s are statistically significantly lower than measures made at 4 deg/s. (**d**) Box and whisker plot indicate little difference in absolute direction-bias across the different test-speeds. (**e**) Switching magnitude plot across speed conditions. There is significant difference in mean SD of DEM-gain for both 8 deg/s and 16 deg/s compared to 4 deg/s. Note that outlier analysis has been applied by removing data (indicated by crosses) that is more than three scaled median absolute deviations (MAD) away from the median.

This discrepancy is likely attributable to the influence of something akin to “response bias” on the reliability of DEM data as compared to subjective report. Figure 9a,b (column 1) plots “V” functions from three of the six CVn participants—who were mis-classified based on data from the low-speed conditions—measured at 4 and 16 deg/s respectively. Note how noisy the DEM-gain data are in Fig. 9a, as indicated by the high mean squared error (MSE) of the fit ‘V’ function, compared to fits in the higher-speed condition (Fig. 9b). Comparing the MSE for all participants across speed (Fig. 9c) showed similar patterns with both 8 and 16 deg/s speed conditions leading to a significantly lower MSE than 4 deg/s. 16 deg/s had by far the lowest mean MSE of 0.11. This finding is not attributable to low-speed conditions eliciting lower DEM-gain; analysis in fact shows that the opposite is true (see Appendix B). Rather note that in Fig. 9a the red and blue symbols are more likely to flip sign around the mean (solid black line), at a given red-green mix, in lower compared to higher speed conditions. Recall that red/blue symbols colour code the direction of the coloured grating (red: left, blue: right) and DEM-response data are signed for colour direction (positive: towards colour-component direction, negative: towards luminance direction). Were the participant to randomly switch between colour and luminance components, (regardless of their contrast) a random distribution of red and blue data-points around the mean gain-level would be noticeable. This can be quantified by taking the SD of the DEM response for all trials moving left (red points), and the SD of the DEM response for all trials moving right (blue points) and averaging the two values (Fig. 9a,b—3rd column). Comparing random switching for all participants across speed (Fig. 9e) shows significantly higher switching at the lower speed.

Another source of variability in the DEM-gain data could arise from a bias towards responding in one direction (left or right) more than in another. When DEM-gain is signed for absolute direction the anticipated mean-gain at a given red-green mix should be zero (since we balanced left and right drift-directions of both colour and luminance components). Were the DEM-data to be biased towards a particular absolute direction then the red and blue data points would form widely separated clusters above and below the mean gain level. We can quantify this by averaging the DEM response signed for absolute direction rather than colour-component direction, as seen in Fig. 9a,b (column 2) for all three CVn participants. Figure 9d compares the direction bias for all participants across speed. Unlike switching, the magnitude of direction bias stays almost constant across speed, with no distinguishable pattern.

Why is switching more frequent at low speeds? Recall that participants were asked to “follow the stimulus,” in an effort to elicit more reliable DEM responses. Having been instructed to continuously track the stimulus, participants who did not know what to follow could voluntarily track and by extension, switch between individual components (colour or luminance) on an arbitrary basis, in low-speed but not in high-speed conditions. For this reason, we recommend the use of higher speed stimuli for DEM testing.

Why didn’t CA91 suffer from this problem? Recall they used a method of adjustment (to null motion) and not a two alternative forced choice, as we did. Is response bias arising from our use of eye movement measures or from our forced choice procedure? To answer this, we reran our test on four of our CVn’s (participant 15, 16, 18, 19) but had them simultaneously make perceptual reports of stimulus direction using the computer keyboard (left and right arrow key to represent the respective direction). Appendix C plots results from both types of measures, which shows DEM or subjective responses lead to similar estimates of response bias. Thus, it is the forced choice procedure (rather than the measure used) that determines the level of response bias.

## Discussion

Adapting an approach described by CA91, we have further developed and validated an automated test for human colour vision deficiency, based entirely on eye movements made in response to dynamic-coloured stimuli. However, unlike CA91 who relied on subjective inputs to measure motion nulls, our use of eye movements creates an entirely objective and involuntary test that requires minimal instructions to administer in clinic (or may even be self-administered at home). Participants CV-status was most accurately determined using a 16 deg/s stimulus drift speed, where our results closely agreed with both the categorisation and measure of severity made using the gold standard anomaloscope. However, unlike the anomaloscope, our test is significantly shorter and simpler to administer, making it fit for use on both young and old participants who may be unable to comply with the anomaloscope (e.g., unable to sit for long durations, have difficulty understanding and following instructions, etc.). Further to this, our test may also be used on nonverbal populations such as babies.

### Comparison to previous work

We report good agreement between results from our objective test of colour vision stats and results from both the clinical gold standard screening procedure (Ishihara plates) and from a diagnostic procedure (the anomaloscope-test, based on patients’ subjective colour judgments). We also compared our results to those from another test (CA91) which uses participants’ subjective judgement of motion direction (rather than the DEM/optokinetic response) for stimuli essentially identical to our own. A notable difference between the results of our study and CA91 is that we find that colour vision status is more reliably determined by data measured in higher speed than in lower speed conditions. This is likely because of the ‘random switching’ discussed above, where lower speed conditions allowed participants to engage in random attentional tracking of either the colour or luminance component, eliciting inconsistent DEM directions for the same stimulus level.

Another difference between findings from the two studies is that we report (for CVn observers) increasing *C*_Eq_ with increasing stimulus-speed (4 deg/s: Avg. *C*_Eq_ = 4.31% vs 16 deg/s: Avg. *C*_Eq_ = 6.72%). This is opposite to CA91’s findings that temporal frequency reduces *C*_Eq._ Interestingly, when Teller and Palmer^31^ used a paradigm similar to our own to evaluate both CVn adults and children, they too report higher *C*_Eq_ with increasing speed. Teller and Palmer report equivalent luminance contrast of ~ 12% at 25 deg/s, similar to CA91’s results for 4 deg/s. This along with our own results suggests that the neural system supporting DEM has higher chromatic sensitivity at higher speeds (potentially leading to better separation between CVn and CVd).

This difference between Teller and Palmer’s and CA91’s study may be attributable to the different motion processing mechanisms engaged. Recall that CA91 asked participants to fixate on a stationary circular marker in the centre of the screen while making subjective reports of direction. On the other hand, Teller and Palmer (and our own study) instructed participants to actively track the stimulus (i.e. eliciting a combination of OKN and smooth pursuit). The difference in eye movements between these two types of tests (fixation vs tracking) may have impacted on *C*_Eq_—a measure of chromatic sensitivity. Prior work by Krauskopf and Li^58^ and Cavanagh^59^ suggests that while a low-level motion processing mechanism such as OKN is driven well only by a luminance-based stimulus, smooth pursuit may be driven by either a luminance or colour-based stimulus. It would then stand to reason that our stimuli (comprised of a luminance and chromatic gratings) activated two distinct motion processing pathways: (a) a low-level optokinetic system driven largely by luminance, (b) a higher level smooth pursuit system driven by the chromatic image-structure.

We hypothesise that smooth pursuit dominated the DEMs made in response to higher speed stimuli, based on the similarity in findings between our study and that of Crognale and Schor^41^. Their study measured consistency of look-OKN (i.e. smooth pursuit) vs stare-OKN for isoluminant chromatic stimuli. They noted irregular stare-OKN for isoluminant chromatic stimuli, similar to the DEM responses we report for our lower speed condition (Fig. 9c, left box and whisker). In contrast Crognale and Schor^41^ reported consistent look-OKN with the same iso-luminant stimuli, similar to the DEM we measured in our higher speed condition (Fig. 9c, right box and whisker). As such the consistency of our OKN estimates measured at higher speeds suggests strong active tracking of the stimulus (over a pure-OKN response). We note that a stronger smooth pursuit response would drive tracking of the colour stimulus (over the luminance stimulus) and would likely increase *C*_Eq_ for CVn’s.

Further work is however needed to test participant response across a wider variety of speed conditions, and to see how average *C*_Eq_ value changes across speed for different colour vision groups.

### Further development

The current test runs 40 × 2.5 s trials to derive an objective estimate of equibrightness and equivalent luminance contrast. Compared to the anomaloscope procedure, our test reduces the time required to diagnose colour vision deficiency by a factor of 10. That DEM’s could be exclusively comprised of involuntary eye movements also means that it can potentially be tested on nonverbal populations such as babies. With that said, there is scope for further improvement.

A significant challenge for a wide-scale role-out of our DEM-based CV test is display calibration. Display calibration is necessary to establish e.g. the physical equiluminance point for a given device, and is typically achieved by making a series of measurements of display luminance using a photometer at different levels of display activation. The validity of this approach is predicated on testing being conducted under similar lighting conditions to the calibration. This may limit the test’s use to clinics where typically the device can be calibrated on-site and lighting conditions under which the device is calibrated are maintained.

At-home calibration would either require a photometer or an observer with ostensibly normal colour vision (for a subjective calibration procedure). Alternatively, at-home testing could make use of pre-calibrated displays or of displays that exhibit high levels of consistency “out of the box” (e.g., iPads). For these options to work under different home lighting conditions, we could potentially analyse environment light (via the webcam) to shift the white point. Home based testing would also require additional data-quality checks to ensure the head and eye positions are reasonably within the limits of the eye tracker/screen. Further research would be needed to gauge the decrease in sensitivity/specificity when this DEM colour vision tests were used in a home-setting without an administrator and varying light conditions.

In terms of additional improvements, DEM responses are variable across participants, and participants that exhibit little to no DEM are often misclassified in the high-speed condition. Implementing open loop OKN where the stimulus follows the eye movements is known to significantly increase the gain^60,61^ and would likely help improve the robustness of the measures.

To reduce test time, we are developing an adaptive procedure^62,63^ that uses current and previous DEM measures to update the R–G mix, in order to more accurately and quickly estimate the motion null points. Reducing test time could be crucial for testing young children who already struggle with the anomaloscope^8^ and are too young to recall numbers used in the Ishihara plates. In-fact previous work has successfully measured OKN in children between 1 and 3 months to estimate equibrightness^64^.

Our automated test is a promising first step towards more accessible, accurate and in-depth colour vision diagnosis in clinics and or homes. Given recent advances in software-based eye tracking running on computers/tablets/phones equipped with front-facing cameras^65^, our test could become a simple, reliable, automated colour vision assessment that could be downloaded for use by clinician and patient alike.

## Supplementary Information

Supplementary Information 1.

Supplementary Video 1.

Supplementary Video 2.
