# Author Correction: Diagnosis of colour vision deficits using eye movements

**DOI:** 10.1038/s41598-022-14563-6

**Published:** 2022-08-02

**Authors:** Aryaman Taore, Gabriel Lobo, Philip R. Turnbull, Steven C. Dakin

**Affiliations:** 1 School of Optometry and Vision Science, The University of Auckland, Auckland, New Zealand; 2 New Zealand National Eye Centre, The University of Auckland, Auckland, New Zealand; 3 UCL Institute of Ophthalmology, University College London, London, UK

Correction to: Scientific Reports https://doi.org/10.1038/s41598-022-11152-5, published online 11 May 2022

The original version of this Article contained an error in Reference 21, which was incorrectly given as:

Screening for color blindness using optokinetic nystagmus*. Investig. Ophthalmol. Vis. Sci.* **25**, 463 (1984).

The correct reference is listed below:

Cavanagh, P., Anstis, S. & Mather, G. Screening for color blindness using optokinetic nystagmus. *Investigative ophthalmology & visual science* **25**, 463–466 (1984).

The original Article has been corrected.

