# Supplementary material for: Diagnosis of colour vision deficits using eye movements
Source: Sci Rep. 2022 May 11;12:7734. doi: 10.1038/s41598-022-11152-5 (PMC9095692; doi:10.1038/s41598-022-11152-5)
Supplement: Supplementary file 1 — Supplementary Information 1. [file 41598_2022_11152_MOESM1_ESM.pdf]

Appendix A

Participant	Gender	Age	Median match (0-73)	Matching range	CV category	Ishihara Plates
**1**	F	18	44.25	4.5	N	P
**2**	F	18	49.25	6.5	N	P
**3**	F	19	45.25	8.5	N	P
**4**	M	19	40.5	5	N	P
**5**	F	20	43	6	N	P
**6**	F	20	43	6	N	P
**7**	F	21	43	4	N	P
**8**	M	21	45.5	9	N	P
**9**	F	21	43	4	N	P
**10**	M	21	47.25	5.5	N	P
**11**	F	21	43.25	3.5	N	P
**12**	F	22	43.5	3	N	P
**13**	F	22	46.75	4.5	N	P
**14**	F	22	43.5	3	N	P
**15**	F	23	42.25	7.5	N	P
**16**	M	23	44	4	N	P
**17**	M	29	46	6	N	P
**18**	M	35	43.25	3.5	N	P
**19**	M	34	44.25	6.5	N	P
**20**	F	52	42	4	N	P
**21**	F	55	43	3	N	P
**22**	F	61	44	4	N	P
**23**	M	65	43	6.5	N	P
**24**	F	17	26	38	D	F
**25**	M	20	24.25	20.5	D	F
**26**	M	22	30.5	31	D	F
**27**	M	24	22.5	15	D	F
**28**	F	30	41.25	17.5	D	F
**29**	M	48	24.5	21	D	F
**30**	M	55	19	34	D	F
**31**	M	64	39	68	D	F
**32**	M	65	24.5	21	D	F
**33**	M	35	45.5	21	P	F
**34**	M	50	41	32	P	F

Appendix A. Participant demographics and matching results from the anomaloscope test. Median match is the median of colour values matched during the anomaloscope test, while matching range is the range of the matches covered. Colour vision (CV) category is coded: N=Normal Trichromat, D=Deuteranope and P=Protanope. The last column indicates participant result for the 14-plate Ishihara; correctly identifying >=12 plates is a Pass (P) and <12 plates is a Fail (F). Results are ordered by age within CV category.

Appendix B

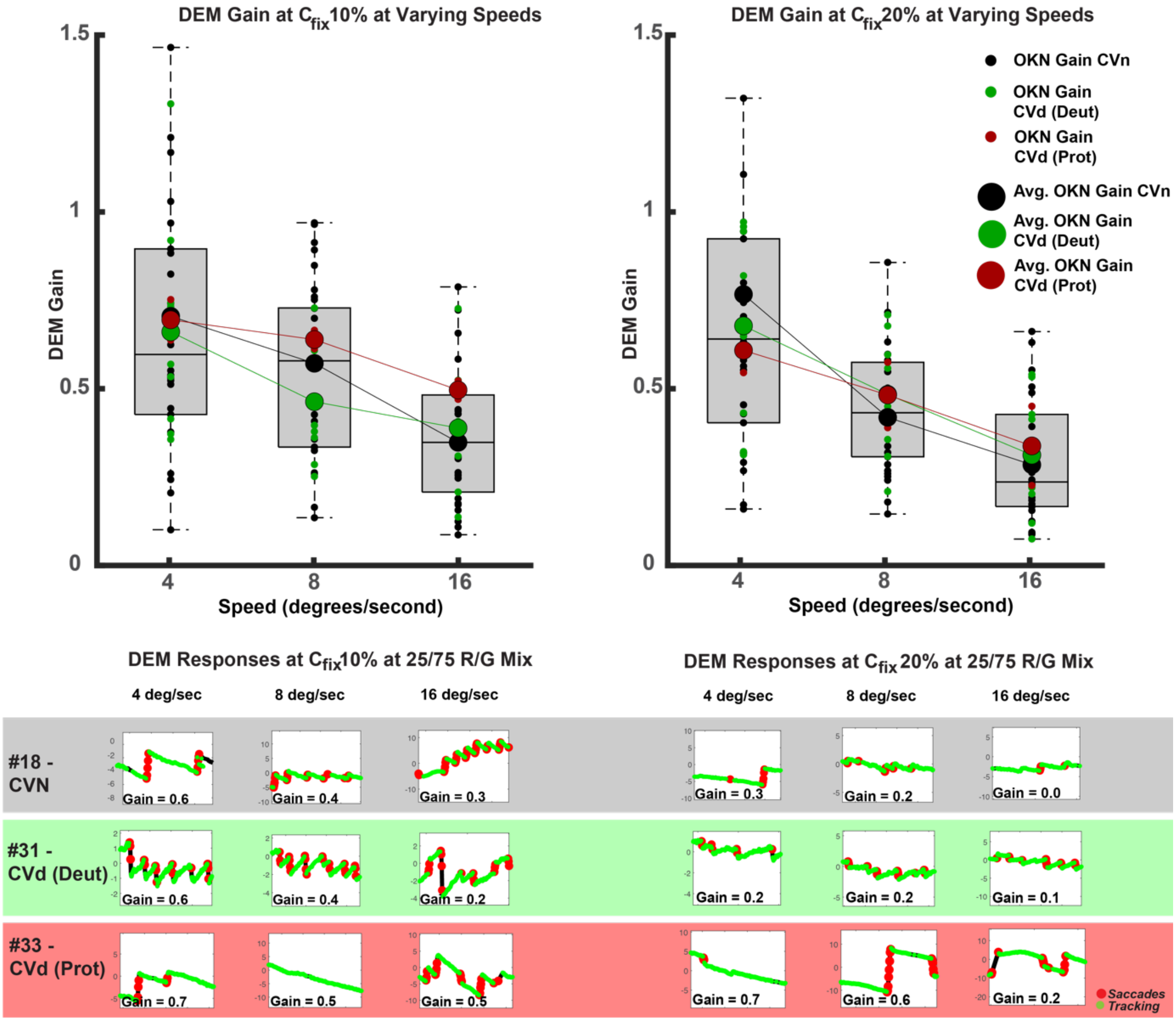

Appendix B. DEM gain for all participants measured in different speed conditions with (a) C_{fix} = 10% and (b) C_{fix} = 20%. Note the decrease in gain with increasing speed. Below the box and whisker plots are typical DEM responses elicited by R20:G80 stimuli for three participants tested with different speed gratings.

Appendix C

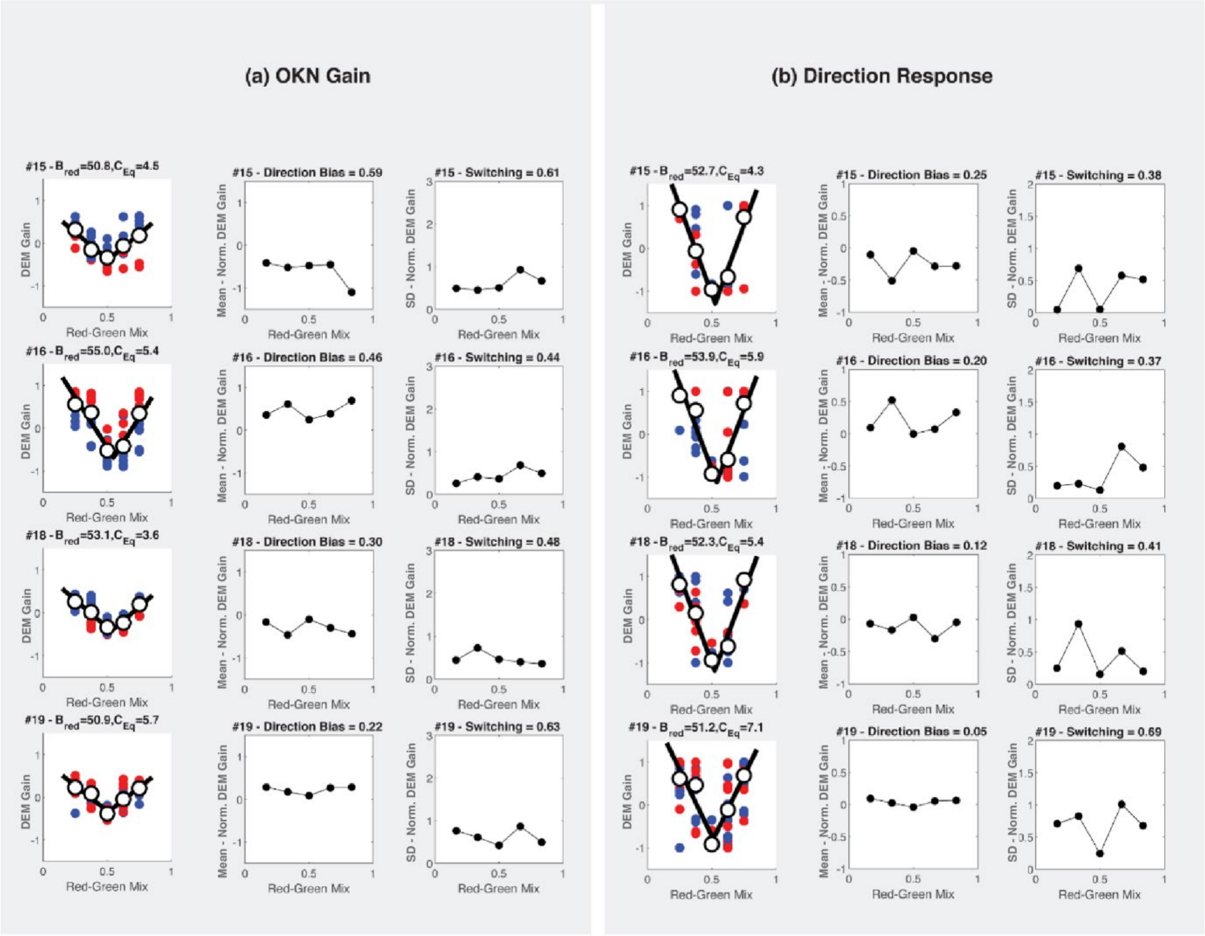

Appendix C. Plot of the fit V functions (1st,4th column) , the corresponding direction bias (2nd,5th column) and the corresponding Switching magnitude (3rd,6th column) for four CVn's tracking our stimulus (C_{fix}=20%, 16 deg/s) with their eyes while making perceptual reports of stimulus direction using the computer keyboard. (a) Plots the measures using the DEM gain. Whereas (b) plots the measures using the key presses (left and right arrow key to represent the respective direction). Note the similarity in Switching, especially for participants 16,18 and 19 regardless of the measure used for deriving the level of response bias.

Video 1

(a,b) Two vertical sine-wave gratings moving in opposite directions are summed to generate the (c) combined stimulus grating. (a) One component grating is defined by a modulation in luminance (generated by in-phase spatial modulation of red and green channels). (b) The other grating is defined by a modulation in chromaticity (generated by anti-phase spatial modulation of red and green channels).

Video 2

(a) Schematic of typical horizontal eye position plot against time in response to the stimulus shown in (c); note the sawtooth pattern consistent with optokinetic nystagmus. (b) A signed measure of response-gain (derived from eye-position) quantifies how consistent tracking is with the chromatic (positive gain) or luminance (negative gain) components of the stimulus. Looking at (a,b) note that as the red-green balance of the grating shifts, observers track first the chromatic component, then the luminance component before finally reverting to the chromatic component. The weak-response/low-gain regions that punctuate periods of tracking are occasions when neither grating component dominates (referred to as *motion nulls*).
